# Phased genome sequence of an interspecific hybrid flowering cherry, ‘Somei-Yoshino’ (*Cerasus* × *yedoensis*)

**DOI:** 10.1093/dnares/dsz016

**Published:** 2019-07-23

**Authors:** Kenta Shirasawa, Tomoya Esumi, Hideki Hirakawa, Hideyuki Tanaka, Akihiro Itai, Andrea Ghelfi, Hideki Nagasaki, Sachiko Isobe

**Affiliations:** 1 Kazusa DNA Research Institute, Japan; 2 Shimane University, Japan; 3 Kyoto Prefectural University, Japan

**Keywords:** floral bud, flowering cherry, interspecific hybrid, phased genome sequence, transcriptome

## Abstract

We report the phased genome sequence of an interspecific hybrid, the flowering cherry ‘Somei-Yoshino’ (*Cerasus* × *yedoensis*). The sequence data were obtained by single-molecule real-time sequencing technology, split into two subsets based on genome information of the two probable ancestors, and assembled to obtain two haplotype phased genome sequences of the interspecific hybrid. The resultant genome assembly consisting of the two haplotype sequences spanned 690.1 Mb with 4,552 contigs and an N50 length of 1.0 Mb. We predicted 95,076 high-confidence genes, including 94.9% of the core eukaryotic genes. Based on a high-density genetic map, we established a pair of eight pseudomolecule sequences, with highly conserved structures between the two haplotype sequences with 2.4 million sequence variants. A whole genome resequencing analysis of flowering cherries suggested that ‘Somei-Yoshino’ might be derived from a cross between *C. spachiana* and either *C. speciosa* or its relatives. A time-course transcriptome analysis of floral buds and flowers suggested comprehensive changes in gene expression in floral bud development towards flowering. These genome and transcriptome data are expected to provide insights into the evolution and cultivation of flowering cherry and the molecular mechanism underlying flowering.

## 1. Introduction

Flowering cherry, called sakura, is Japan’s unofficial national flower and is a popular ornamental tree in Japan and elsewhere. Cherry blossoms are symbols of spring, when blooming typically occurs. Accordingly, flowering cherries are important resources for the tourism industry in the spring season in Japan. More than 200 cultivars of flowering cherry are grown.^1^ The nomenclature and, in particular, the genus name (*Prunus* or *Cerasus*) has been under discussion. We use the genus name *Cerasus* in accordance with recent molecular and population genetic analyses.^2^ Most cultivars belong to a species complex with ten basic diploid founders (2*n* = 16), *C. apetala*, *C. campanulata*, *C. incisa*, *C. jamasakura*, *C. leveilleana*, *C. maximowiczii*, *C. nipponica*, *C. sargentii*, *C. spachiana* and *C. speciosa*. Recently, a new species, *C. kumanoensis*, has been described from Japan.^3^

‘Somei-Yoshino’ (*C.* × *yedoensis*), also known as Yoshino cherry, is the most popular cultivar of flowering cherry. ‘Somei-Yoshino’ is believed to have been originated in a nursery in the Somei area of Edo (the former name of Tokyo), followed by its spread throughout Japan. ‘Somei-Yoshino’ is probably derived from an interspecific hybrid between two diploids (2*n* = 16),^4^*C. spachiana* and *C. speciosa.*^5–7^ An alternative hypothesis is that ‘Somei-Yoshino’ arose from a cross between *C. spachiana* and a hybrid of *C. jamasakura* and *C. speciosa.*^8^ It is self-incompatible, like other members of the Rosaceae, and accordingly no seeds are produced by self-pollination. Even if self-pollinated seeds are obtained, genotypes would be segregated owing to the high heterozygosity. Therefore, ‘Somei-Yoshino’ is clonally propagated by grafting or cutting and distributed. The clonality is supported by DNA analyses.^6^^,^^9^ Thus, the taxonomic classification has been well investigated. However, to the best of our knowledge, there are few studies of the molecular mechanism underlying flowering in flowering cherry to date, despite extensive analyses of other members of the Rosaceae.

‘Some-Yoshino’ trees are used as standards for forecasting the flowering date of cherry blossoms in the early spring every year. Bud breaking and flowering are important and scientifically intriguing growth stages. In buds, the floral primordia are generally initiated in the summer (late June–August), after which the primordia start to differentiate into floral organs. After differentiation is completed, the buds enter a dormancy period during the winter. Recent studies have evaluated the molecular mechanisms underlying dormancy release as well as flowering in fruit tree species belonging to the Rosaceae.^10^^,^^11^ Phytohormones and transcriptional regulators involved in dormancy initiation and release have been characterized, including gibberellic acids (GAs) and abscisic acid (ABA). *DELLA* genes, containing a conserved DELLA motif involved in GA signalling, and *CBF/DREB1* (C-repeat-binding factor/dehydration-responsive element-binding factor 1) genes involved in cold acclimation have been analysed in apple (*Malus* × *domestica*)^12^^,^^13^ and Japanese apricot (*Prunus mume*).^14^ The involvement of ethylene signalling, perhaps via crosstalk with ABA, has also been discussed based on a study of *EARLY BUD-BREAK 1* (*EBB1*), which encodes an AP2 type/ethylene-responsive transcription factor.^12^*DORMANCY-ASSOCIATED MADS-BOX* (*DAM*) genes in the same family as *SHORT VEGETATIVE PHASE* (*SVP*) genes,^15^^,^^16^*FLOWERING LOCUS T* (*FT*) and *CENTRORADIALIS* (*CEN*)/*TERMINAL FLOWER 1* (*TFL1*), encoding PEBP-like proteins involved in floral initiation and meristem development, are involved in dormancy.^17^ These previous studies provide insight into the genetic basis of dormancy and flowering in fruit tree species belonging to the Rosaceae.

Genetic and genomic analyses are straightforward approaches to gain insights into the flowering mechanism in cherry blossoms. Whole genome sequences of >100 plant species have been published.^18^ Usually, the targets are haploids or inbred lines to simplify the genomic complexity. However, advanced long-read sequencing technologies and bioinformatics methods have made it possible to determine the sequences of complex genomes.^19–21^ For example, an assembly strategy for single-molecule real-time sequencing data has been developed to generate phased sequences in heterozygous regions of F1 hybrids.^22^ Furthermore, chromosome-scale phased genome assemblies for F1 hybrids have been obtained by linked read sequencing technology, providing long-range genome information,^23^ or by single-molecule real-time sequencing combined with Hi-C data.^24^^,^^25^ Haplotype-resolved sequences have been obtained for F1 cattle by a trio-binning strategy in which genome sequences with allelic variation are resolved before assembly.^26^

In this study, to obtain insights into the molecular mechanisms underlying cherry blossom flowering, we conducted genome and transcriptome analyses of the interspecific hybrid ‘Somei-Yoshino’. The genome sequence of another interspecific hybrid flowering cherry, *C.* × *nudiflora*, formerly named *Prunus yedoensis* var. *nudiflora*,^2^ has been published.^27^ However, all genomic regions derived from the two different progenitor species (*C. spachiana* and *C. jamasakura*) are totally collapsed. Therefore, we established the phased genome sequence of *C.* × *yedoensis*, ‘Somei-Yoshino’, representing the two genomes of the probable progenitors (*C. spachiana* and *C. speciosa*). Using the genome sequences as a reference, a time-course transcriptome analysis of ‘Somei-Yoshino’ floral buds and flowers, with a special focus on dormancy and flowering-related genes, was also conducted to characterize the physiological changes during flowering.

## 2. Materials and methods

### 2.1. Plant materials

A ‘Somei-Yoshino’ tree grown in Ueno Park (Tokyo, Japan) was used for genome assembly. This tree, i.e. #136, is presumed to be the original according to a polymorphism analysis of three genes and its location.^7^^,^^28^ In addition, 139 trees, including a ‘Somei-Yoshino’ clone maintained at Shimane University (SU), Shimane, Japan and five trees of wild species (*C. campanulata*, *C. pseudocerasus*, *C. sargentii*, *C. speciosa* and *Padus grayana*), were used for a clustering analysis (Supplementary Table S1). An F1 mapping population, YSF1, was produced by hand pollination between Yama-Zakura (*C. jamasakura*) and a clone of ‘Somei-Yoshino’ as a female and male parent, respectively, both of which are planted at the Kazusa DNA Research Institute (KDRI), Chiba, Japan. The ‘Somei-Yoshino’ clones at SU and KDRI were used for the transcriptome analysis.

### 2.2. Clustering analysis of genetically divergent cultivars

Genomic DNAs of the 139 trees were extracted from young leaves using the DNeasy Plant Mini Kit (Qiagen, Hilden, Germany) and double-digested with the restriction enzymes *Pst*I and *Eco*RI. ddRAD-Seq libraries were constructed as described previously^29^ and sequenced using the Illumina HiSeq2000 (San Diego, CA, USA) to obtain 93 bp paired-end reads. Low-quality reads were trimmed using PRINSEQ v. 0.20.4^30^ and adapter sequences were removed using fastx_clipper (parameter, -a AGATCGGAAGAGC) in FASTX-Toolkit v. 0.0.13 (http://hannonlab.cshl.edu/fastx_toolkit (9 July 2019, date last accessed)). The high-quality reads were mapped onto genome sequences of either sweet cherry (*Cerasus avium*; formerly named *Prunus avium*),^31^ Japanese apricot (*P. mume*)^32^ or peach (*Prunus persica*)^33^ using Bowtie2 v. 2.2.3.^34^ Biallelic SNPs were called from the mapping results using the mpileup command in SAMtools v. 0.1.19,^35^ and low-quality SNPs were removed using VCFtools v. 0.1.12b^36^ with the following criteria: including only sites with a minor allele frequency of ≥0.05 (--maf 0.05), including only genotypes supported by ≥5 reads (--minDP 5), including only sites with a quality value of ≥999 (--minQ 999) and excluding sites with ≥50% missing data (--max-missing 0.5). A dendrogram indicating a simple classification of the cultivars based on the SNPs was constructed using the neighbour-joining method implemented in TASSEL 5^37^ and population structure was investigated using ADMIXTURE v. 1.3.0 with default settings (*K* = 1–20).^38^

### 2.3. Assembly of the ‘Somei-Yoshino’ genome

Genomic DNA was extracted from young leaves of ‘Somei-Yoshino’ tree #136 using the DNeasy Plant Mini Kit (Qiagen). A paired-end sequencing library (insert size of 500 bp) and three mate-pair libraries (insert sizes of 2, 5 and 8 kb) were constructed using the TruSeq PCR-free Kit (Illumina) and Mate-pair Kit (Illumina), respectively, and sequenced using the MiSeq and HiSeqX platforms (Illumina). The size of the ‘Somei-Yoshino’ genome was estimated using Jellyfish v. 2.1.4.^39^ High-quality reads after removing adapter sequences and trimming low-quality reads as described above were assembled using SOAPdenovo2 v. 1.10^40^ (parameter: -K 121). Gaps, represented by Ns in the sequence, were filled with high-quality paired-end reads using GapCloser v. 1.10^40^ (parameter: -p 31). The resultant sequences were designated CYE_r1.0.

High-molecular-weight DNA was extracted from young leaves of ‘Somei-Yoshino’ tree #136 using Genomic Tip (Qiagen) to prepare the SMRTbell library (PacBio, Menlo Park, CA, USA). The sequence reads obtained from the PacBio Sequel system were assembled using FALCON-Unzip^22^ to obtain an assembly, CYE_r2.0. Furthermore, the PacBio reads were divided into two subsets using the TrioCanu module of Canu v. 1.7,^26^ in which Illumina short reads of two probable ancestors of ‘Somei-Yoshino’, i.e. *C. spachiana* ‘Yaebeni-shidare’ and *C. speciosa* Ohshima-zakura, were employed. Each subset of data was assembled and polished using FALCON assembler v. 2.1.2,^41^ in which parameters were: pa_HPCdaligner_option = -v -B128 -e0.75 -M24 -l1500 -k14 -h70 -w8 -s100 -t14; and ovlp_HPCdaligner_option = -v -B128 -M24 -k24 -h1024 -e.96 -l2500 -s100. The two assemblies were designated CYEspachiana_r3.0 and CYEspeciosa_r3.0, and were combined to obtain CYE_r3.0, representing the ‘Somei-Yoshino’ genome. Assembly completeness was evaluated using BUSCO v. 3.0.2,^42^ for which Plants Set was employed as data sets, and a mapping rate analysis of whole genome sequence data for ‘Somei-Yoshino’ reads to the references was performed (see below for details).

### 2.4. Genetic map construction and pseudomolecule establishment

Genomic DNA was extracted from the ovules of YSF1 seeds using the Favorgen Plant Kit (Ping-Tung, Taiwan) and digested with *Pst*I and *Eco*RI to construct the ddRAD-Seq library. The library was sequenced on the Illumina NextSeq platform. High-quality reads were mapped onto CYEspaciana_r3.0 and CYEspeciosa_r3.0 using Bowtie2 v. 2.2.3.^34^ Biallelic SNPs were called from the mapping results using the mpileup command in SAMtools v. 0.1.19,^35^ and low-quality SNPs were deleted using VCFtools v. 0.1.12b^36^ with the criteria used for the clustering analysis described above. The SNPs from the two references were merged, grouped and ordered using Lep-Map3 v. 0.2.^43^ Flanking sequences of the SNP sites (100 bases up- and downstream of the SNPs) were compared with the genome sequence of sweet cherry, PAV_r1.0,^31^ by BlastN with a cutoff value of 1E-40. Probable misassemblies found in the mapping process were broken, and the resultant sequence set was designated CYE_r3.1. According to map positions, the CYE_r3.1 sequences were oriented and assigned to the genetic map of ‘Somei-Yoshino’ to establish pseudomolecule sequences. Sequence variation between the two pseudomolecule sequences, CYEspaciana_r3.1 and CYEspeciosa_r3.1, was detected using the show-snps function of MUMMER v. 3.23,^44^ for which outputs from NUCmer were employed. In parallel, the genome structure of CYE_r3.1_pseudomolecule was compared with those of sweet cherry, peach, Japanese apricot and apple using D-GENIES.^45^

### 2.5. Gene prediction and annotation

Total RNA was extracted from 12 stages of buds within 1 month in 2017 as well as from leaves, stems, sepals, petals, stamens and carpels. RNA-Seq libraries were prepared using the TruSeq Stranded mRNA Sample Preparation Kit (Illumina) and sequenced by MiSeq. The obtained reads were mapped to the CYE_r3.1 sequences to determine gene positions using TopHat2 v. 2.0.14.^46^ The positional information was used in BREAKER2 v. 2.1.0^47^ to gain training data sets for AUGUSTUS v. 3.3^48^ and GeneMark v. 4.33.^49^ The two training sets and a preset of SNAP v. 2006-07-28 for *Arabidopsis* as well as peptide sequences of sweet cherry (v1.0.a1), peach (v2.0.a1) and apple (GDDH13 v1.1) registered in the Genome Database for Rosaceae^50^ and those of Japanese apricot^32^ were analysed using MAKER pipeline v. 2.31.10^51^ to predict putative protein-coding genes in the CYE_r3.1 sequences. Genes annotated using Hayai-Annotation Plants v. 1.0^52^ (with a sequence identity threshold of 80% and query coverage of 80%) were selected as a high-confidence gene set.

### 2.6. Gene clustering, multiple sequence alignment and divergence time estimation

Potential orthologues were identified from genes predicted in seven genomes (two genomes of ‘Somei-Yoshino’ and one each of sweet cherry, Japanese apricot, peach and apple, as well as *Arabidopsis* as an outgroup) using OrthoMCL v. 2.0.9.^53^ The single copy orthologues in the seven genomes were used to generate a multiple sequence alignment using MUSCLE v. 3.8.31,^54^ in which indels were eliminated by Gblocks v. 0.91b.^55^ A maximum-likelihood algorithm based phylogenetic tree showing the evolutionary process of the species was constructed from the alignments with the Jones–Taylor–Thornton model in MEGA X v. 10.0.5.^56^ The divergence time was calculated using MEGA X v. 10.0.5^56^ assuming that the divergence time between apple and peach was ∼34 to 67 MYA in TIMETREE.^57^

### 2.7. Repetitive sequence analysis

A database of repeat sequences of the ‘Somei-Yoshino’ genome was established using RepeatModeler v. 1.0.11.^58^ The repeat database as well as that registered in Repbase^59^ was used to predict repetitive sequences in CYE_r3.1 using RepeatMasker v. 4.0.7.^60^

### 2.8. Whole genome resequencing analysis

Genomic DNA of eight representative trees of the SU collection and one of the parental trees of the mapping population, ‘Yama-Zakura’, were digested with NEBNext dsDNA Fragmentase (New England BioLabs, Ipswich, MA, USA) for whole genome shotgun library preparation using the Illumina TruSeq PCR-free Kit. The sequences were determined on the Illumina NextSeq platform. Read trimming, read mapping to the CYE_r3.1 sequence and SNP identification were performed as described above. Effects of SNPs on gene functions were evaluated using SnpEff v. 4.2.^61^

### 2.9. Transcriptome analysis

Additional RNA-Seq libraries were prepared from buds at 24 stages collected in 2017 at KDRI and in 2014 and 2015 at SU using the TruSeq Stranded mRNA Library Prep Kit (Illumina) and sequenced on the NextSeq500 (Illumina). High-quality reads after removing adapter sequences and trimming low-quality reads as mentioned above were mapped to the pseudomolecule sequences of CYE_r3.1 using HISAT2 v. 2.1.0,^62^ and reads on each gene model were quantified and normalized to determine FPKM values using StringTie v. 1.3.5^63^ and Ballgown v.2.14.1^64^ in accordance with the protocol paper.^65^ The R package WGCNA v.1.66^66^ was used for network construction and module detection.

## 3. Results

### 3.1. Clustering analysis of cherry cultivars

We obtained ∼1.9 million (M) high-quality reads per line after trimming adapters and low-quality sequences from the ddRAD-Seq library. The reads were mapped onto the genome sequences of sweet cherry (PAV_r1.0), Japanese apricot and peach (v1.0) with mapping alignment rates of 70.8%, 77.8% and 68.7%, respectively (Supplementary Table S2). We detected 46,278 (sweet cherry), 31,973 (Japanese apricot) and 33,199 (peach) high-confidence SNPs. A clustering tree based on the 46,278 SNPs and a population structure analysis indicated that the cherry collection consisting of 139 trees was derived from at least eight founders (*K* = 8) (Supplementary Fig. S1). The result suggested that the ‘Somei-Yoshino’ genome consisted of *C. spachiana* and *C. speciose* genomic features.

### 3.2. Assembly of the ‘Somei-Yoshino’ genome

The ‘Somei-Yoshino’ genome size was estimated by a *k*-mer analysis with 14.3 Gb of paired-end reads (20.7×) obtained by MiSeq (Supplementary Table S3). The distribution of distinct *k*-mers (*k* = 17) showed two peaks at multiplicities of 18 and 37, indicating heterozygous and homozygous regions, respectively (Supplementary Fig. S2). This result suggested that the heterozygosity of the ‘Somei-Yoshino’ genome was high. In other words, ‘Somei-Yoshino’ is likely an interspecific hybrid harboring components of two different genomes. The total size of the two genomes was ∼690 Mb.

Totals of 132.5 Gb of paired-end reads (192× genome coverage) and 69.1 Gb of mate-pair data (100×) (Supplementary Table S3) were assembled into 1.2 million scaffold sequences. The total length of the resultant scaffolds, i.e. CYE_r1.0, was 686.9 Mb, including 63.6 Mb of Ns with an N50 length of 142.5 kb (Supplementary Table S4). Only 62.3% of complete single copy orthologues in plant genomes were identified in a BUSCO analysis (Supplementary Table S4). Paired-end reads of ‘Somei-Yoshino’ (20.7×) were mapped onto CYE_r1.0 with a mapping rate of 76.6%. We found that 82.4% of SNPs were homozygous for the reference type. Ideally, both rates should be close to 100% if the assembly was fully extended and the two genomes were separated, or phased. Distributions of the sequence depth of coverage showed a single peak at the expected value of 21× (Supplementary Fig. S3). When we mapped the reads to the sequence of *C.* × *nudiflora* (Pyn.v1),^27^ two peaks at 22× (expected) and 44× (double the expected value) were observed (Supplementary Fig. S3), indicating a mixture of phased and unphased sequences.

To extend the sequence contiguity and to improve the genome coverage, PacBio long-read technology was employed to obtain 37.3 Gb of reads (54×) with an N50 read length of 17 kb (Supplementary Table S3). The long reads were assembled using FALCON-Unzip into 3,226 contigs [470 primary contigs (488 Mb) and 2,756 haplotigs (116 Mb)] spanning a total length of 605.4 Mb with an N50 length of 2.3 Mb, i.e. CYE_r2.0 (Supplementary Table S4). A BUSCO analysis indicated that 97.0% of complete BUSCOs (9.1% single copy and 87.9% duplicated, as expected) were represented in the assembly (Supplementary Table S4). The mapping rate of the ‘Somei-Yoshino’ reads was 95.3%, and 97.1% of SNPs were homozygous for the reference type. Most of the sequences were phased, with one major peak of genome coverage at 21× (Supplementary Fig. S3); however, the total length was 13% shorter than the estimated size and no haplotype information was available.

We used a trio-binning approach to obtain the entire sequences of the two haplotype sequences. The long reads (37.3 Gb, 54×) were divided into two subsets based on whole genome resequencing of the two trees, i.e. Cerasus_1-43 (*C. spachiana*, ‘Yaebeni-shidare’) and Cerasus_1-71 (*C. speciose*, Ohshima-zakura). The resultant subsets included 18.9 and 18.2 Gb for *C. spachiana* and *C. speciosa*, respectively, and 0.3 Mb of unassigned reads. The subsets were separately assembled to obtain 2,281 contigs (717 primary contigs and 1,564 associated contigs including duplicated repetitive sequences) covering 350.1 Mb, i.e. CYEspachiana_r3.0, and 2,271 contigs (800 primary contigs and 1,471 associated contigs) covering 340.0 Mb, i.e. CYEspeciosa_r3.0 (Supplementary Table S4). The total sequence (i.e. CYE_r3.0) spanned 690.1 Mb and consisted of 4,552 contigs with an N50 length of 1.0 Mb (Supplementary Table S4). The complete BUSCO score for CYE_r3.0 was 96.8% (10.6% single copy and 86.2% duplicated, as expected), while those for CYEspachiana_r3.0 and CYEspeciosa_r3.0 were 90.9% (69.3% single copy and 21.6% duplicated) and 88.9% (72.1% single copy and 16.8% duplicated), respectively (Supplementary Table S4). The mapping rate of the ‘Somei-Yoshino’ reads was as high as 96.3%, and 96.2% of SNPs were homozygous for the reference type. The sequence depth of coverage was distributed as expected, with a single peak at 20× (Supplementary Fig. S3). In addition, the mapping rate of the ddRAD-Seq reads of the 139 trees was improved to be 90.2% (Supplementary Table S2). Therefore, CYE_r3.0 was used for further analyses because it satisfied all of the established criteria.

### 3.3. Genetic map for ‘Somei-Yoshino’

Approximately 2.0 million high-quality ddRAD-Seq reads per sample were obtained from YSF1 and mapped to either CYEspachiana_r3.0 or CYEspeciosa_r3.0 with alignment rates of 79.3% and 80.3%, respectively (Supplementary Table S5). We detected 16,145 and 17,462 SNPs from the alignments with the references of CYEspachiana_r3.0 and CYEspeciosa_r3.0, respectively. Of these, 23,532 heterozygous SNPs in ‘Somei-Yoshino’ were used for a linkage analysis. The SNPs were assigned to eight groups and ordered, covering 458.8 cM with 16,933 SNPs in 694 genetic bins (Supplementary Tables S6 and S7). The map was split into two for CYEspachiana_r3.0 and CYEspeciosa_r3.0, covering 448.9 cM with 8,280 SNPs (628 genetic bins) and 446.3 cM with 8,653 SNPs (645 genetic bins), respectively. The genetic bins were common for 579 loci on the two maps, suggesting that the sequences in the common bins were the same loci. A comparison of the genetic maps with the genome sequence of sweet cherry, PAV_r1.0 (Supplementary Fig. S4), indicated a high similarity of the genome structures in the two species.

### 3.4. Genetic anchoring of the assemblies to the chromosomes

**Figure 1:**
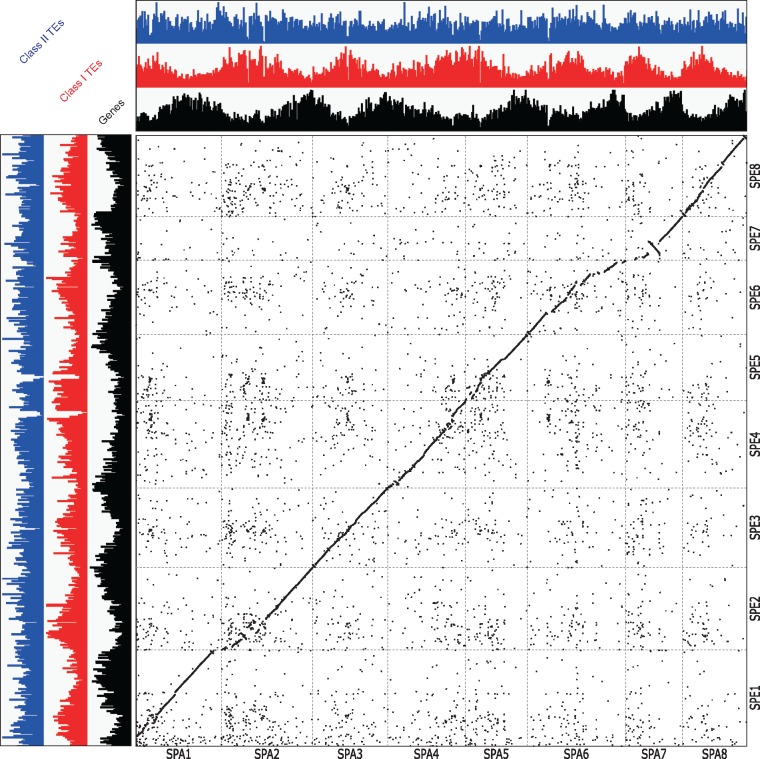
Synteny of the two haplotype pseudomolecule sequences of the ‘Somei-Yoshino’ genome. *X*- and *Y*-axes are sequences of CYE_r3.1spachiana (SPA1 to 8) and CYE_r3.1speciosa (SPE1 to 8), respectively. Densities of genes, retrotransposons (Class I) and DNA transposons (Class II) were indicated by bar plots in black, red and blue, respectively.

In the genetic mapping process, we found 19 potential misassemblies in 18 contig sequences of CYE_r3.0. The contigs were broken between SNPs mapped to different linkage groups. Finally, we obtained 4,571 contigs with an N50 length of 918.2 kb and the same total length (690.1 Mb). This final version of contigs was named CYE_r3.1, consisting of CYEspachiana_r3.1 (2,292 contigs, N50 length of 1.2 Mb) and CYEspeciosa_r3.1 (2,279 contigs, N50 length of 800.6 kb) (Table 1). Of these, 184 CYEspachiana_r3.1 contigs (221.8 Mb) and 262 CYEspeciosa_r3.1 contigs (199.2 Mb) were assigned to the genetic maps (Supplementary Table S8). The contigs were connected with 10,000 Ns to establish the ‘Somei-Yoshino’ pseudomolecule sequences consisting of 4,571 contigs covering 418 Mb. The structures of the two pseudomolecule sequences were well conserved with a few exceptions in chromosomes 2, 4 and 5 (Fig. 1). We observed 2,371,773 and 2,392,937 sequence variants, including SNPs and indels, in CYEspachiana_r3.1 (one variant every 93 bp) and CYEspeciosa_r3.1 (one variant every 83 bp), respectively, of which 0.4% were deleterious mutations (Supplementary Table S9). The structure of the ‘Somei-Yoshino’ genome showed high synteny with the genomes of other members of Rosaceae (Supplementary Fig. S5).

**Table 1:** Assembly statistics of the final version of the ‘Somei-Yoshino’ genome sequence

	CYE_r3.1 (total)	CYEspachiana_r3.1	CYEspeciosa_r3.1
Number of contigs	4,571	2,292	2,279
Total length (bases)	690,105,700	350,135,227	339,970,473
Contig N50 (bases)	918,183	1,151,237	800,562
Longest contig (bases)	11,102,098	11,102,098	6,718,036
Gap length (bases)	0	0	0
GC (%)	37.9	37.8	38.1
Number of predicted genes	95,076	48,280	46,796
Mean size of genes (bases)	966	975	951

### 3.5. Gene prediction and annotation

We initially predicted 222,168 putative genes using the MAKER pipeline. All genes were annotated by a similarity search against the UniProtKB database using the Hayai-Annotation Plants pipeline to select 94,776 non-redundant high-confidence genes. Then, 300 genes showing sequence similarity to genes involved in flowering and dormancy in the Rosaceae (Supplementary Table S10) were manually added. A total of 95,076 genes (48,280 and 46,796 from CYEspachiana_r3.1 and CYEspeciosa_r3.1, respectively) were selected as a high-confidence gene set for CYE_r3.1 (Table 1), preferentially located on the distal ends of the pseudomolecule sequences (Fig. 1). The total length of the coding sequences was 91.9 Mb (13.3% of the CYE_r3.1) with an N50 length of 1,512 bases and a GC content of 44.8%. This gene set included 94.9% complete BUSCOs (12.8% single copy and 82.1% duplicated). Out of the 95,076 genes, 26,463 (27.8%), 34,996 (36.8%) and 46,502 (48.9%) were assigned to Gene Ontology slim terms in the biological process, cellular component and molecular function categories, respectively. Furthermore, 3,972 genes had enzyme commission numbers.

We found two pairs of self-incompatible genes, *S* determinants for pollen (*S*-RNase) and pistils (SFB: *S* haplotype-specific F-box); CYE_r3.1SPE0_g058440.1 (S-RNase) and CYE_r3.1SPE0_g058430.1 (SFB) were *S* genes of the *PyS1* haplotype, and CYE_r3.1SPE0_g046700.1 (S-RNase) and CYE_r3.1SPE0_g046660.1 (SFB) were *S* genes of *PyS2*. For dormancy, we detected a cluster of six *DAM*-like genes, as reported in the Japanese apricot genome,^32^ in the pseudomolecule sequence of SPA1 (CYE_r3.1SPA1_g039840.1 to CYE_r3.1SPA1_g039890.1). In addition, *CBF* gene clusters were also found in SPA5 (CYE_r3.1SPA5_g014520.1 to CYE_r3.1SPA5_g014610.1) and SPE5 (CYE_r3.1SPE5_g016380.1 to CYE_r3.1SPE5_g016430.1).

### 3.6. Gene clustering and divergence time of ‘Somei-Yoshino’ ancestors

The 95,076 predicted genes were clustered with those of apple, sweet cherry, Japanese apricot, peach and *Arabidopsis* to obtain 29,091 clusters, involving 36,396 and 35,559 genes from CYEspachiana_r3.1 and CYEspeciosa_r3.1, respectively (Supplementary Table S11). While 15,849 clusters including 29,251 and 29,020 genes of CYEspachiana_r3.1 and CYEspeciosa_r3.1, respectively, were shared with the two ancestor genomes (namely, core gene clusters), 4,083 (7,145 genes of CYEspachiana_r3.1) and 3,558 (6,539 genes of CYEspeciosa_r3.1) clusters were generated in only one genome (i.e. genome-specific gene clusters). The remaining 5,601 clusters were absent from the ‘Somei-Yoshino’ genome but presented in either the five members.

**Figure 2:**
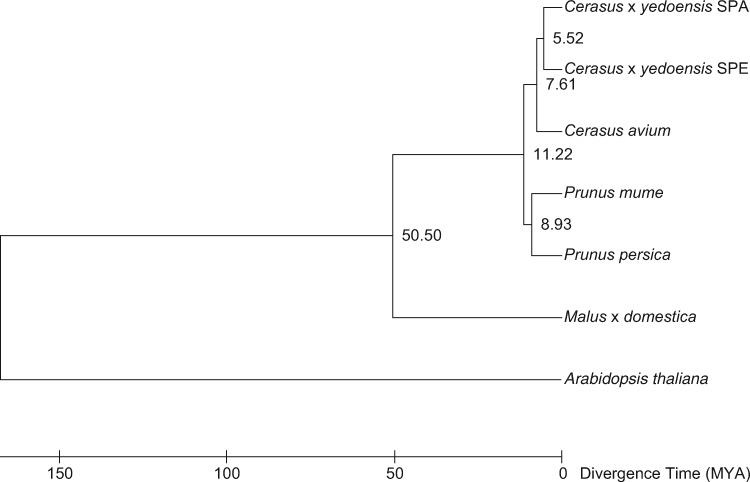
Phylogenetic tree indicating the divergence time of ‘Somei-Yoshino’. The two genomes of ‘Somei-Yoshino’ are indicated by SPA and SPE, representing CYEspachiana_r3.0 and CYEspeciosa_r3.0, respectively. Divergence times (MYA; million years ago) between branches are shown.

However, 8,125 clusters were common across the seven tested genomes, and 1,254 clusters consisting of one gene from each genome were selected for divergence time estimation. When the divergence time between apple and peach was set to 34– 67 MYA,^57^ the divergence time between the two haplotype sequences of ‘Somei-Yoshino’ was set to 5.52 MYA (Fig. 2).

### 3.7. Repetitive sequence analysis

A total of 293.3 Mb (42.5%) of CYE_r3.1 (690.1 Mb) was identified as repetitive sequences, including transposable elements (Supplementary Table S12), which occupied 142.9 Mb (40.8%) and 150.4 Mb (44.2%) of CYEspachiana_r3.1 and CYEspeciosa_r3.1, respectively. The most prominent repeat types were long-terminal repeat retrotransposons (104.0 Mb; 14.1%), e.g. *Gypsy*- and *Copia*-types, followed by DNA transposons (65.1 Mb; 8.8%). While the DNA transposons were distributed over the genome evenly, the retroelements were located in the middle of the chromosomes where are probably centromeric and pericentromeric heterochromatin regions (Fig. 1). The retrotransposon-rich regions were corresponded to the disrupted regions of chromosomes 2, 4 and 5 in the synteny analysis (Fig. 1).

### 3.8. Whole genome resequencing analysis

Approximately 136 million high-quality whole genome sequence reads was obtained from eight representatives in a population structure analysis (Supplementary Table S13) and the parents of the mapping population, Yama-Zakura and ‘Somei-Yoshino’. In addition, 250 million sequence reads of *C.* × *nudiflora*^27^ (SRA accession number SRX3900230) was also employed. The reads were aligned to CYE_r3.1 as a reference with a mapping rate of 88.0%, on average. From the alignment data, we detected 2,307,670 SNPs and 169,664 indels, including 658,873 SNPs and 42,286 indels (28.3%) in CYEspachiana_r3.1 and 1,648,797 SNPs and 127,378 indels (71.7%) in CYEspeciosa_r3.1. Of these, 8,872 SNPs (0.4%) were deleterious mutations (Supplementary Table S14).

In Somei-Yoshino, the reads were evenly mapped to the references of CYEspachiana_r3.1 (48.7%) and CYEspeciosa_r3.1 (47.6%) (Supplementary Fig. S6). Most of the loci (94.5% of SNPs in CYEspachiana_r3.1 and 96.9% in CYEspeciosa_r3.1) were homozygous for the reference type, as expected (Supplementary Fig. S7). Only 61.7% and 52.9% of SNPs in *C.* × *nudiflora* were reference-type homozygotes on CYEspachiana_r3.1 and CYEspeciosa_r3.1, respectively (Supplementary Fig. S6), and read mapping rates were 52.2% (CYEspachiana_r3.1) and 39.8% (CYEspeciosa_r3.1) (Supplementary Fig. S7).

In Cerasus_1-43 (*C. spachiana*, ‘Yaebeni-shidare’), 69.8% of the reads were preferentially mapped to CYEspachiana_r3.1 (Supplementary Fig. S6), and 80.1% of SNPs detected in CYEspachiana_r3.1 were homozygous for the reference type (Supplementary Fig. S7). In Cerasus_1-71 (*C. speciose*, Ohshima-zakura), 61.1% of reads were mapped to CYEspeciosa_r3.1 (Supplementary Fig. S6) and 73.5% of SNPs in CYEspeciosa_r3.1 were homozygous for the reference type (Supplementary Fig. S7). In the remaining seven cultivars, mapping rates on CYEspeciosa_r3.1 were higher than those on CYEspachiana_r3.1, as in Cerasus_1-71 (*C. speciose*, Ohshima-zakura) (Supplementary Fig. S6).

### 3.9. Transcriptome analysis of flowering dates

RNA-Seq reads were obtained from 12 stages of buds collected every month from May 2014 to April 2015 (Supplementary Table S15) as well as from the 12 stages from 2 to 34 days before anthesis in 2017 used for gene prediction. After trimming, the reads as well as those for the six organs used in the gene prediction analyses were mapped to CYE_r3.1 with a mapping rate of 67.6%, on average. Among the 95,076 predicted genes, 72,248 (76.0%) with a variance across samples of ≥1 were selected. A WGCNA analysis was performed with the expression data for the 24 buds to generate 31 highly co-expressed gene clusters, referred to as modules (Supplementary Fig. S8). The modules were roughly grouped into three main classes expressed in the previous year of flowering, within 1 month, and within 1 week (Supplementary Fig. S9). There were no significant differences in expression patterns of genes of the core and genome-specific clusters (Supplementary Table S16).

**Figure 3:**
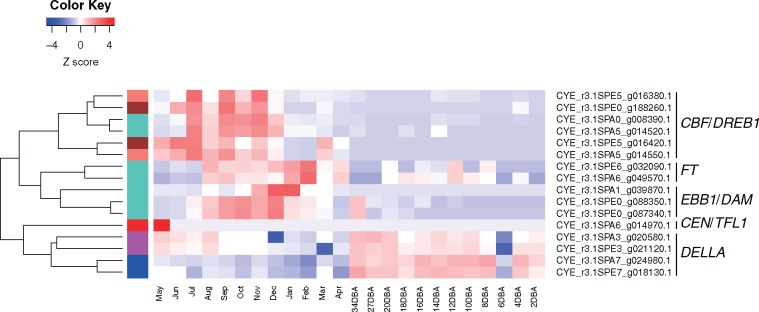
Heat map representing expression patterns of dormancy and flowering genes in ‘Somei-Yoshino’ buds. Colours in each block represent a continuum of gene expression levels with *Z*-score-transformed FPKM (low-to-high gene expression levels are represented by blue to red). May to April are the months and 34DBA to 2DBA are days before anthesis when bud samples were collected. Gene modules based on WGCNA (see also Supplementary Fig. S8) are shown as coloured bars between the dendrogram and heat map.

Based on the literature and databases for Rosaceae, we identified dormancy- and flowering-associated genes [i.e. *DELLA*, *CBF*/*DREB1*, *EBB1*, *DAM* (*SVP*), *FT* and *CEN*/*TFL1* genes]. We detected 35 predicted genes in the ‘Somei-Yoshino’ genome, 16 of which were expressed in ≥1 sample. The expression patterns basically agreed with those of the modules and could be roughly classified into five groups (Fig. 3). The first group (blue and magenta gene modules in Supplementary Fig. S8) consisted of four genes homologous to *DELLA* genes. Their expression levels were elevated in the floral buds ∼1 month before anthesis; expression was also observed in young vegetative buds. The second group (turquoise, brown and salmon gene modules) was highly expressed in the summer and autumn (from July to November) in the floral buds. Six genes homologous to *CBF*/*DREB1* belonged to this group; however, these were classified into three different clusters on the dendrogram. The third group (turquoise gene module) consisted of two *EBB1* homologues and one *DAM* (*SVP*) homologue; these genes were highly expressed in the autumn and winter (from October to December). In the fourth group (turquoise gene module), genes were highly expressed in the winter 2–3 months before anthesis and were homologous to *FT* genes. The fifth group (red gene module) solely included *CEN*/*TFL1*-like genes specifically expressed in vegetative state buds before flower differentiation.

## 4. Discussion

We obtained the genome sequence of the flowering cherry ‘Somei-Yoshino’. To the best of our knowledge, this is the first report of a phased genome sequence of an interspecific hybrid in the Rosaceae or in the kingdom of Plantae, broadly, although genome sequences have been reported for several species belonging to Rosaceae.^50^ Although the genome of another interspecific hybrid cherry flower, *C.* × *nudiflora*, has been reported,^27^ the two homoeologous ancestral genomes (*C. spachiana* and *C. jamasakura*) are totally collapsed, as indicted by the double peaks of sequence depth (Supplementary Fig. S3), resulting in a short assembly size (323.8 Mb). The genome complexity of interspecific hybrids could be compared with those of polyploids and highly heterozygous species. Genome sequences of polyploids and F1 hybrids have been obtained^22^^,^^23^ by single-molecule real-time sequencing technology, linked read sequencing, optical maps and Hi-C.^19–21^ These technologies to obtain phased genome assemblies are limited by haplotype switching,^25^ where two haplotypes are patched to make mosaic genome sequences.

We employed the trio-binning technique^26^ to determine haplotype phases before assembly. This technique was initially developed to construct phased genome sequences of an F1 hybrid between cattle subspecies. Since sequence reads of two sub-genomes were divided into two subsets according to the sequences of the parents, haplotype switching is avoidable. We applied the trio-binning technique to the interspecific hybrid cherry tree. We verified the quality and accuracy of the resultant assembly, CYE_r3.0, by a BUSCO analysis (Supplementary Table S4), the mapping rates of ‘Somei-Yoshino’ reads and ddRAD-Seq reads of the 139 trees to the assemblies (Supplementary Fig. S6 and Supplementary Table S2), and SNP genotypes detected in the mapping results. In addition, the genetic map (Supplementary Tables S6 and S7) and a comparative analysis of the pseudomolecule sequences between the two phased sequences and to those of Rosaceae (Fig. 1 and Supplementary Figs S4 and S5) also supported the quality and accuracy of the assembly. The results of this study suggested that the trio-binning strategy is useful for determining phased genome sequences for highly heterozygous genomes of interspecific hybrids.

Our genome data provided insight into the progenitors of ‘Somei-Yoshino’. Our results were consistent with the conclusions of Baek et al.,^27^ who found that ‘Somei-Yoshino’, *C.* × *yedoensis*, is distinct from a variety in Jeju Island, Korea, *C.* × *nudiflora*. In this study, a population structure analysis suggested that ‘Somei-Yoshino’ was established by two founders, *C. spachiana* and *C. speciosa* (Fig. 2, Supplementary Fig. S1), as suggested in previous studies.^5^^,^^6^ In a whole genome resequencing analysis, sequence reads of Cerasus_1-43 (*C. spachiana*, ‘Yaebeni-shidare’) were preferentially mapped to SPA sequences (Supplementary Fig. S6), and genotypes of most SNPs were homozygous for the reference type (Supplementary Fig. S7). This suggested that the sequence similarity of *C. spachiana* ‘Yaebeni-shidare’ and CYEspachiana_r3.1 was high and therefore that *C. spachiana* can be a candidate parent. While reads of Cerasus_1-71 (*C. speciose*, Ohshima-zakura) were mapped to CYEspeciosa_r3.1 sequences (Supplementary Fig. S6), the frequency of SNP genotypes homozygous for the reference type was not as high as that for Cerasus_1-43 (*C. spachiana*, ‘Yaebeni-shidare’) (Supplementary Fig. S7). This observation might suggest that *C. speciosa* is not an actual parent of ‘Somei-Yoshino’^8^; however, more individual trees of *C. spachiana* and *C. speciose* should be tested to verify the hypotheses. ‘Somei-Yoshino’ genome data can be used in future studies of the origin to determine the most likely parents.

In the clustering analysis, we used SNPs detected from ddRAD-Seq reads mapped on the sweet cherry genome sequence. This result could be improved by using the ‘Somei-Yoshino’ genome sequence as a reference, in which mapping rate was certainly risen up to 90.2% (Supplementary Table S2). Further improvement could be achieved with more trees to cover broaden genetic diversity of flowering cherries including multiple individuals of a single species and distant relatives. In the current dendrogram (Supplementary Fig. S1), distant species, e.g. *C. campanulata*, *C. pseudocerasus*, *C. avium* and *P. grayana*, belonged to a single class. This result disagreed with the actual taxonomy classification.^8^ This bias would be caused by the elimination of rare alleles mostly observed in the few distant species out of the tested plants, since we set the minor allele frequency of 5% as a cut-off value. This threshold value is often used in population genetics,^37^ but might lead mis-classification of cultivated and domesticated organisms, which are often generated by interspecific hybridizations. Indeed, flowering cherry cultivars also potentially include interspecific hybrids, which origins have been sometimes unknown.^2^ Adequate compositions of genome admixture proportions based on prior knowledge on the materials could break this limitation.

We obtained a number of predicted genes. Transcriptome data for the developing bud provided a comprehensive overview of genes expressed during dormancy and flowering processes (Fig. 3). Our analysis was based on previous studies of key genes and fundamental molecular mechanisms underlying dormancy.^10^^,^^11^ Despite some discrepancies, the gene expression patterns observed in our study were generally consistent with previously observed patterns in deciduous fruit tree species in Rosaceae, suggesting that the transcriptome data from this study might be reliable even though biological and technical replicates were lacked. The relatively high expression levels of *DELLA* genes observed at 1 month before anthesis corresponded to the time at which the bud typically transitions from endodormancy to ecodormancy.^14^ GA signalling may reactivate bud development internally at the ecodormancy stage.^67^ The relatively high expression levels of *CBF*/*DREB1* in the summer and decreased expression levels towards the winter is consistent with a role in cold acclimation, as previously reported in almond.^68^ We detected one *DAM* gene that was highly expressed in dormant buds in the winter, in agreement with previous reports^69^; however, two *EBB1* genes, assigned to the same module as *DAM* genes, showed different expression patterns from those in apple and poplar, in which the genes exhibit sharp increases in expression before bud breaking.^12^^,^^13^ This inconsistency may be explained by differences in regulatory mechanisms underlying bud breaking. *FT* genes showed elevated expression levels in buds in February, when endodormancy is almost completed. In addition to the function of floral induction, unknown functions of *FT* genes during dormancy are possible. Interestingly, transgenic plum (*Prunus domestica*) with overexpressed poplar *FT* (*PtFT1*) does not enter a state of endodormancy upon cold treatment or, alternatively, has no chilling requirement after dormancy is established.^70^ Further studies of the role of *FT* genes in dormancy are needed. *CEN*/*TFL1* was highly expressed only in vegetative buds before floral initiation. This observation was consistent with other previous results for species in the Rosaceae.^71^^,^^72^ Our transcriptome data for flowering cherry successfully revealed the comprehensive changes in gene expression during floral bud development towards flowering. The expression patterns of above genes in this study and supposed regulation network for dormancy release of woody plants^10^^,^^73^^,^^74^ are jointly summarized in Supplementary Fig. S10. The transcriptome data set provides a basis for further research aimed at identifying additional genes involved in floral bud development and flowering. Especially, identifying genes involved in the regulation of flowering under *FT* gene (protein) signalling and GA signalling processes is intrigued, and those may be able to utilize for accurate forecasting the flowering date of cherry blossoms.

The genome and transcriptome data obtained in this study are expected to accelerate genomic and genetic analyses of flowering cherry. Owing to the complicated genomes, it is necessary to build additional *de novo* assemblies for divergent flowering cherries, which is a challenging task. Genome-graph-based pan-genome analyses could be used to characterize the complex genomes.^75^ The ‘Somei-Yoshino’ genome sequence would be a resource for the flowering cherry pan-genome analyses. It may provide insights into the evolution and cultivation of flowering cherry as well as the molecular mechanism underlying flowering traits in the species and in the Rosaceae, and it may guide the future cultivation and breeding of flowering cherry.

## Supplementary Material

dsz016_Supplementary_Data
