# Supplementary material for: Phased genome sequence of an interspecific hybrid flowering cherry, ‘Somei-Yoshino’ (Cerasus × yedoensis)
Source: DNA Res. 2019 Jul 23;26(5):379–89. doi: 10.1093/dnares/dsz016 (PMC6796508; doi:10.1093/dnares/dsz016)
Supplement: dsz016_Supplementary_Data [file dsz016_supplementary_data.zip › dsz016-Suppl_data/Supplementary_Figures.pdf]

Supplementary information

Phased genome sequence of an interspecific hybrid flowering cherry, 'Somei-Yoshino' (*Cerasus* × *yedoensis*)

Kenta Shirasawa[1]*, Tomoya Esumi[2], Hideki Hirakawa[1], Hideyuki Tanaka[2], Akihiro Itai[3], Andrea Ghelfi[1], Hideki Nagasaki[1], Sachiko Isobe[1]

[1]Kazusa DNA Research Institute, Japan, [2]Shimane University, Japan, and [3]Kyoto Prefectural University, Japan

*Correspondence:
Kenta Shirasawa
shirasaw@kazusa.or.jp

Supplementary Table S1 Genetically divergent lines of flowering cherry used in this study
Supplementary Table S2 Sequence reads for the ddRAD-Seq analysis of flowering cherry lines
Supplementary Table S3 Number of whole genome shotgun reads for 'Somei-Yoshino'
Supplementary Table S4 Assembly statistics for the 'Somei-Yoshino' genome
Supplementary Table S5 Sequence reads in the ddRAD-Seq analysis for the F1 mapping population
Supplementary Table S6 Genetic map of 'Somei-Yoshino'
Supplementary Table S7 SNP loci on the genetic map
Supplementary Table S8 Summary of the 'Somei-Yoshino' pseudomolecule sequence
Supplementary Table S9 Number of sequence variants between the two genomes of 'Somei-Yoshino' and impacts on gene functions
Supplementary Table S10 Genes underlying flowering and dormancy in the Rosaceae
Supplementary Table S11 Gene clusters based on genes in six species in the Rosaceae and *Arabidopsis thaliana*
Supplementary Table S12 Repetitive sequences in the 'Somei-Yoshino' genome
Supplementary Table S13 Number of whole genome shotgun reads of genetically divergent lines of flowering cherry
Supplementary Table S14 Number of sequence variants among 11 genetically divergent lines
Supplementary Table S15 Number of RNA-Seq reads for 'Somei-Yoshino'
Supplementary Table S16 Number of WGCNA module genes in gene clusters

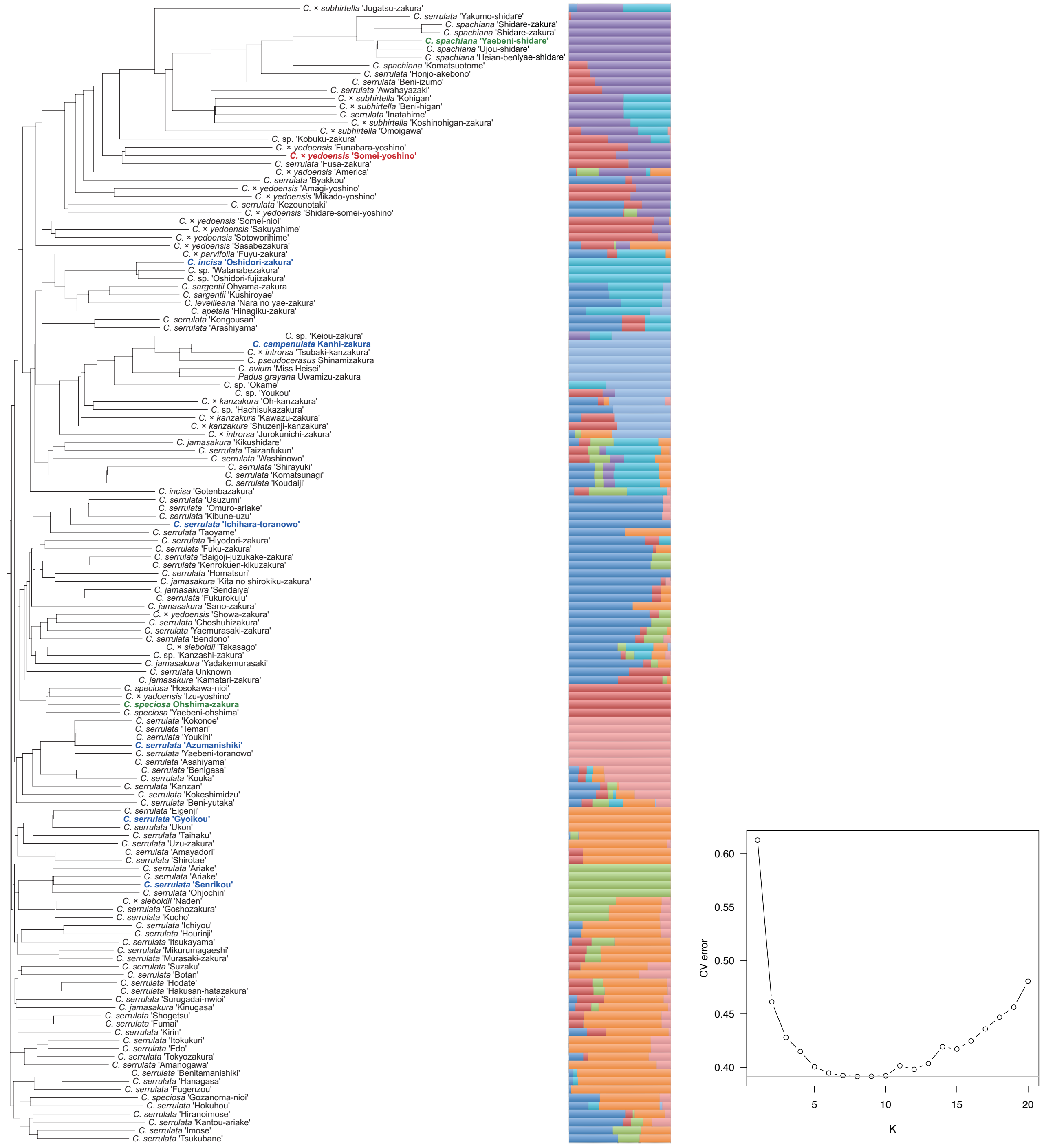

Supplementary Figure S1. Dendrogram and population structure analysis of flowering cherries

The dendrogram is based on genetic distances calculated by the neighbor-joining method. 'Somei-Yoshino' is shown in red, and varieties selected for whole genome resequencing are in blue, including two possible ancestral lines shown in green. Line chart indicates cross-validation errors in the admixture analysis. The best fit model is K = 8.

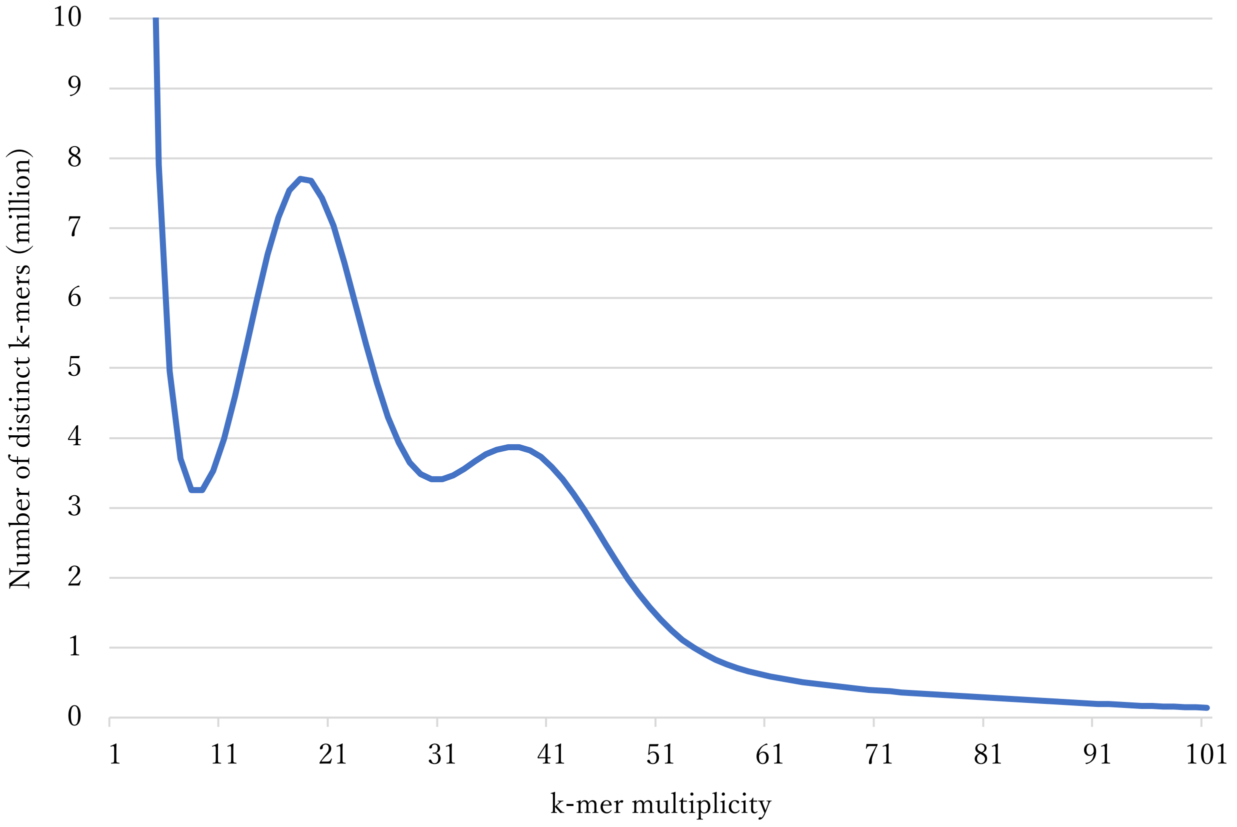

Supplementary Figure S2. Genome size estimation for 'Somei-Yoshino' with the distribution of the number of distinct k-mer (k = 17) with the given multiplicity values

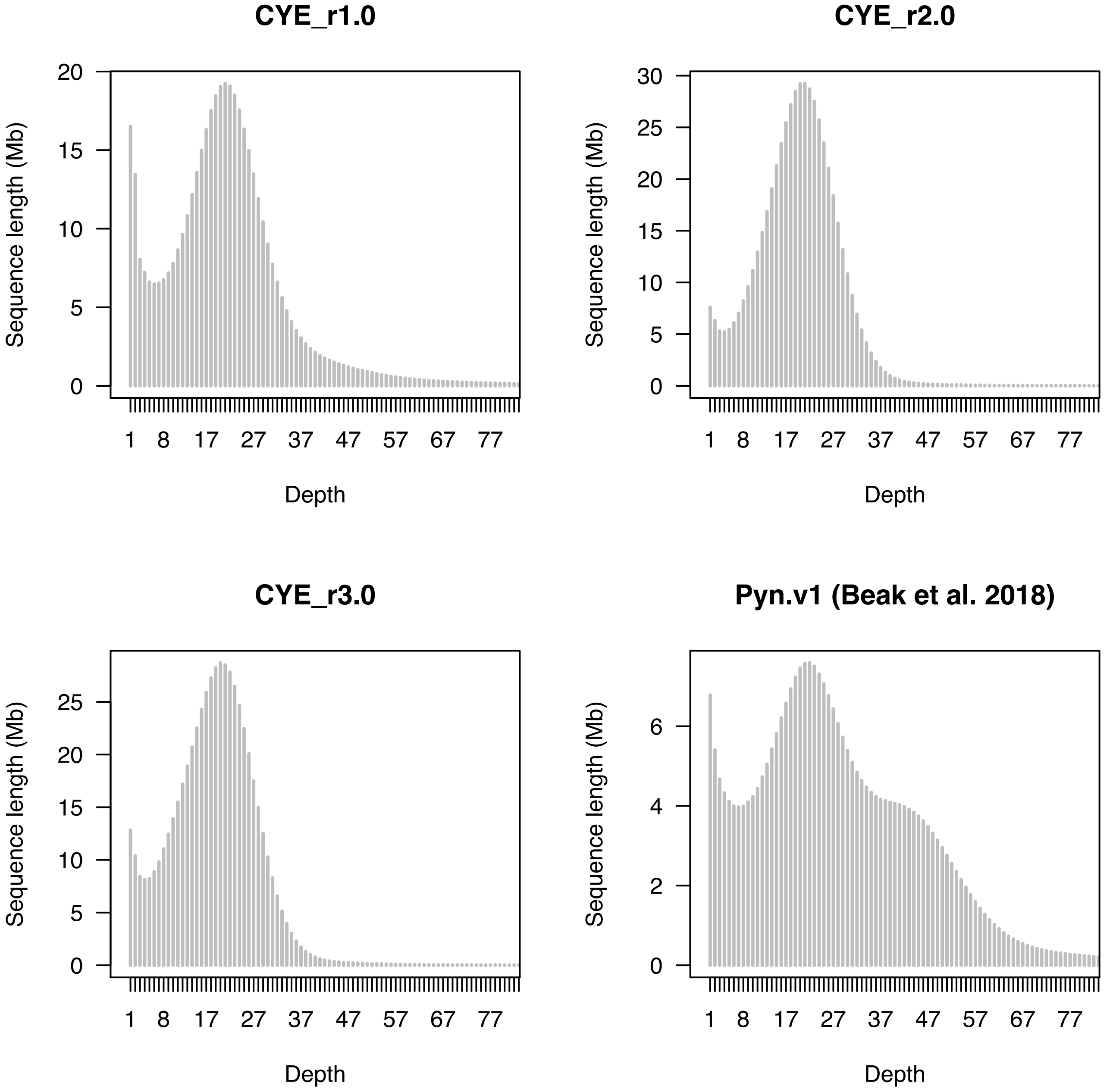

Supplementary Figure S3. Distribution of depths of 'Somei-Yoshino' reads on genome assemblies

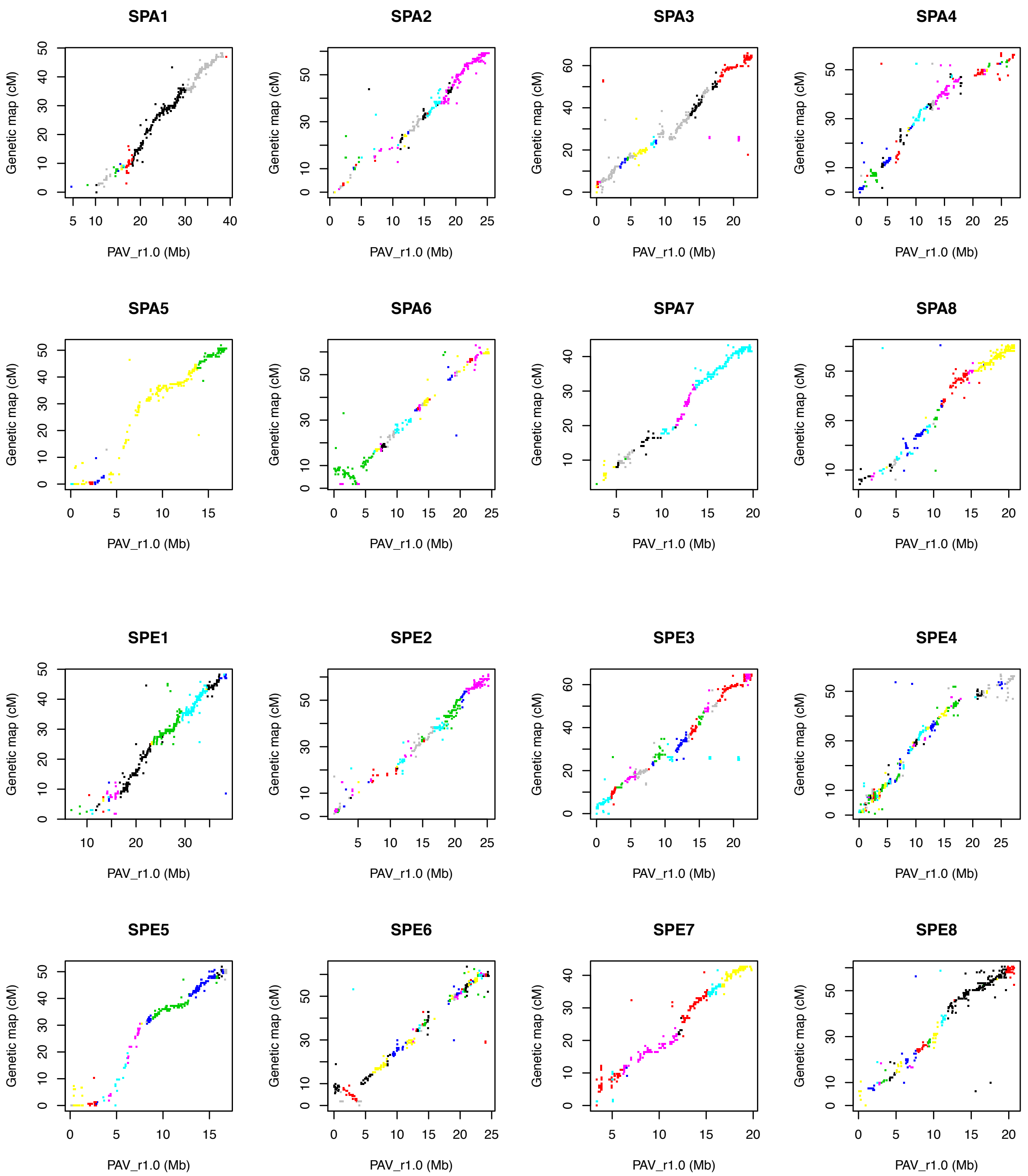

Supplementary Figure S4. Comparative analysis of the 'Somei-Yoshino' genetic maps and the sweet cherry physical maps

Colors indicate contigs of the 'Somei-Yoshino' genome sequences.

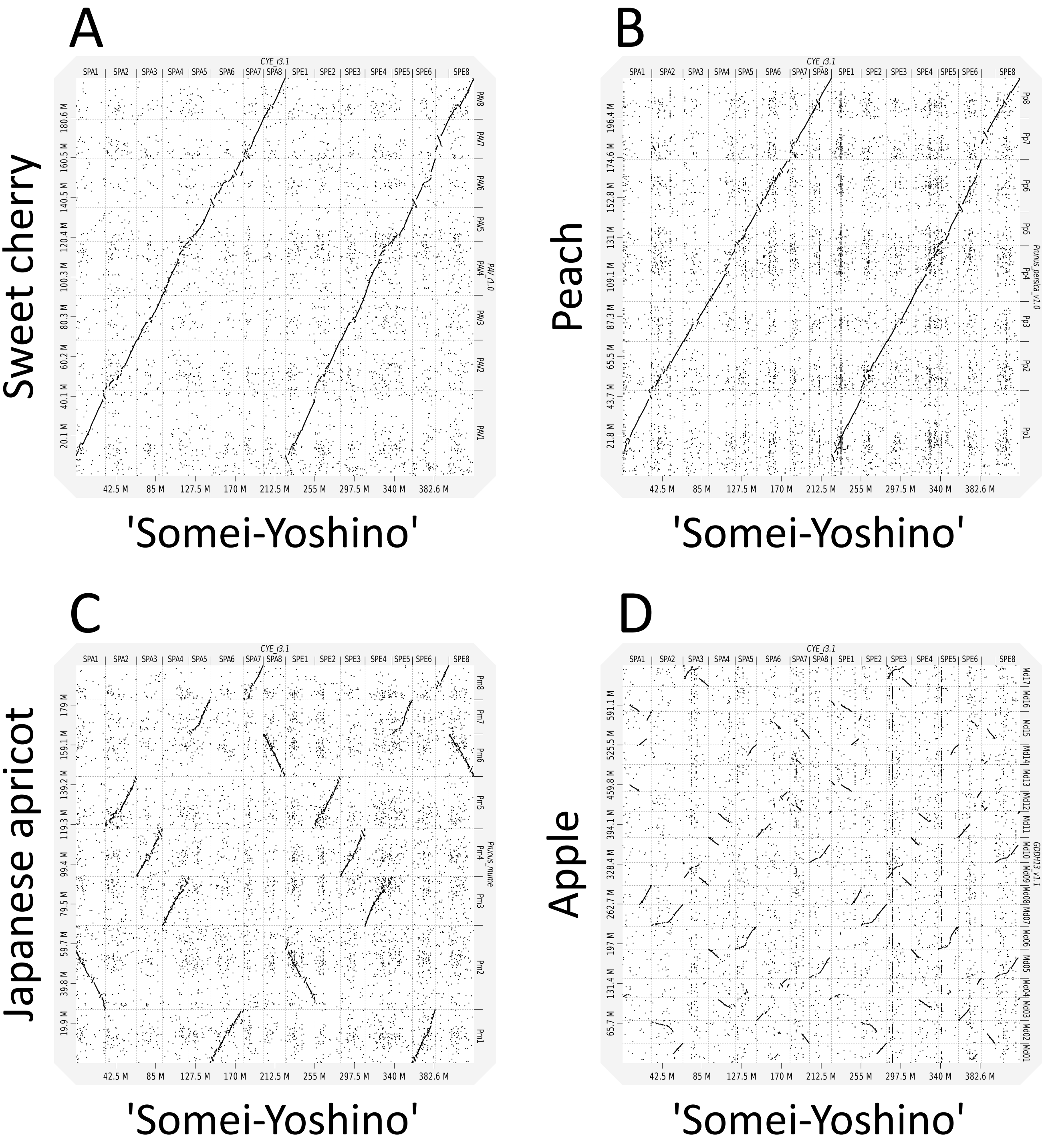

Supplementary Figure S5. Synteny of the 'Somei-Yoshino' pseudomolecule sequences with those of taxa in the Rosaceae

X- and *Y*-axes are sequences of Somei-Yoshino, *C.* × *yedoensis* (SPA1 to 8 and SPE1 to 8) and sweet cherry (A), peach (B), Japanese apricot (C), or apple (D).

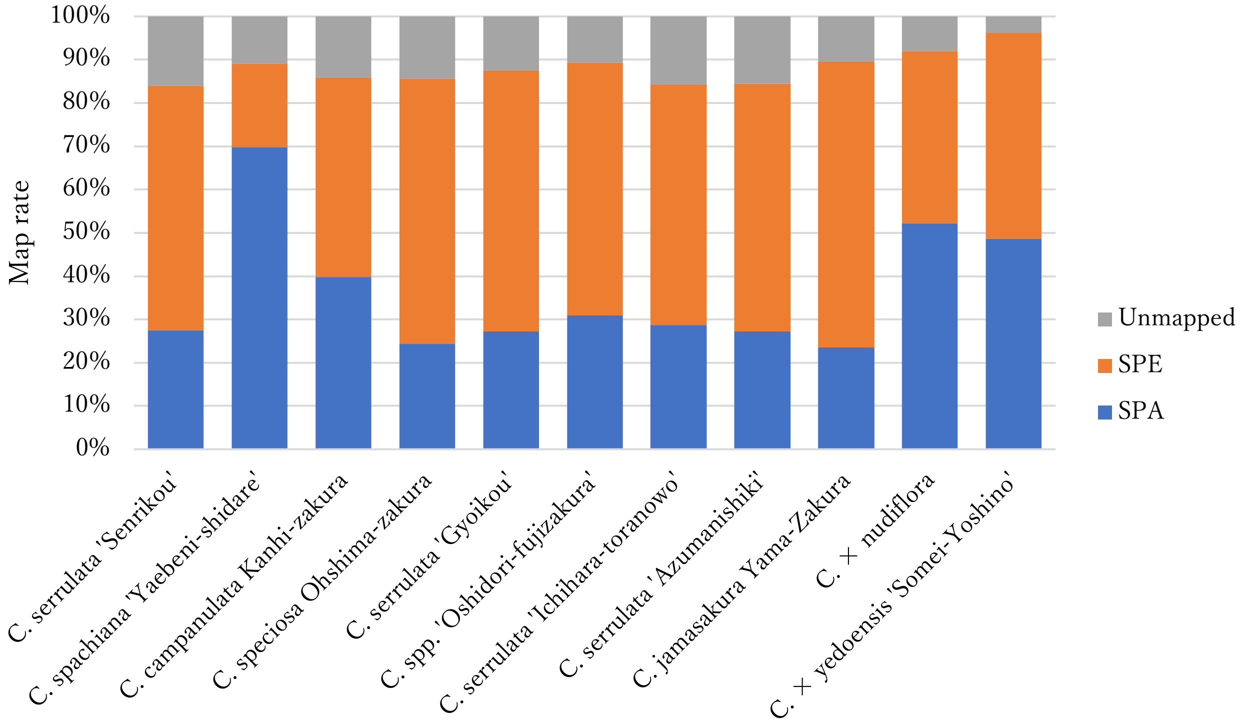

Supplementary Figure S6. Mapping rate of resequencing reads on the 'Somei-Yoshino' genome sequence Reads are classified into three groups: mapped to CYE_r3.1spachiana (SPA) and CYE_r3.1speciosa (SPE) and unmapped.

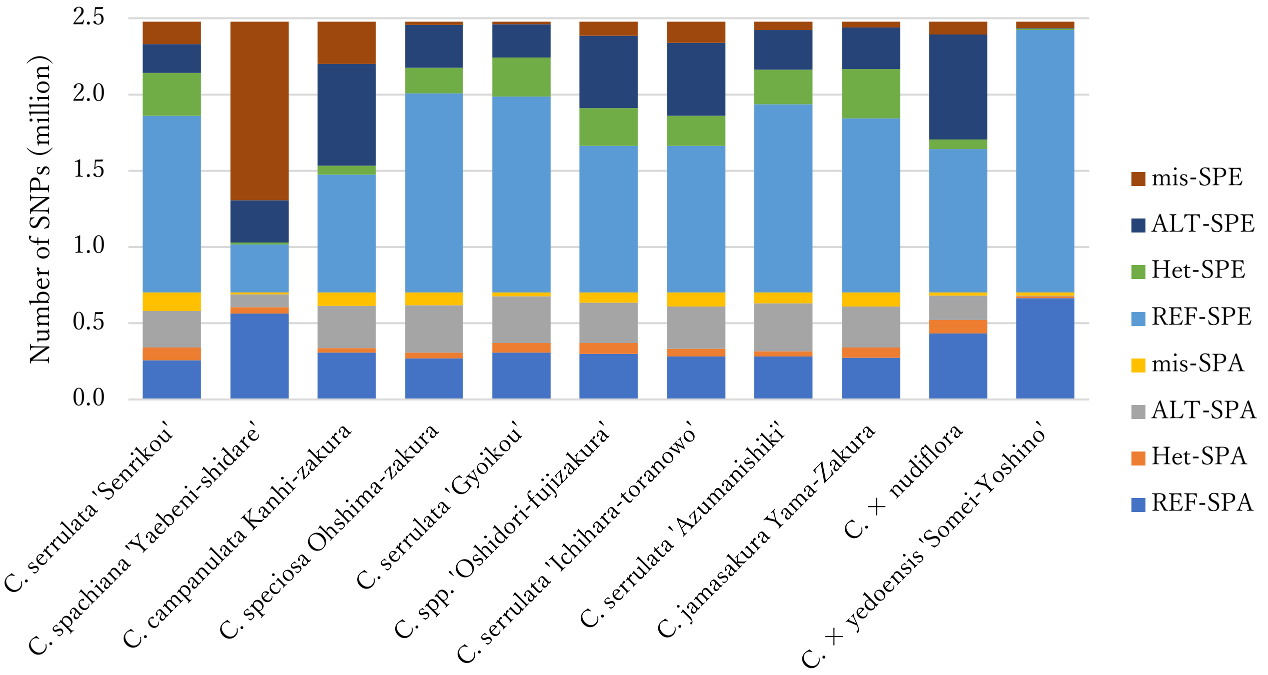

Supplementary Figure S7. Number of SNP genotypes with respect to 'Somei-Yoshino' as a reference Homozygous SNPs as reference-type alleles and alternative-type alleles are indicated by REF and LAT, respectively. Heterozygous SNPs are denoted Het, and missing data are shown as mis. SNPs on CYE_r3.1spachiana and CYE_r3.1speciosa are indicated by SPA and SPE, respectively.

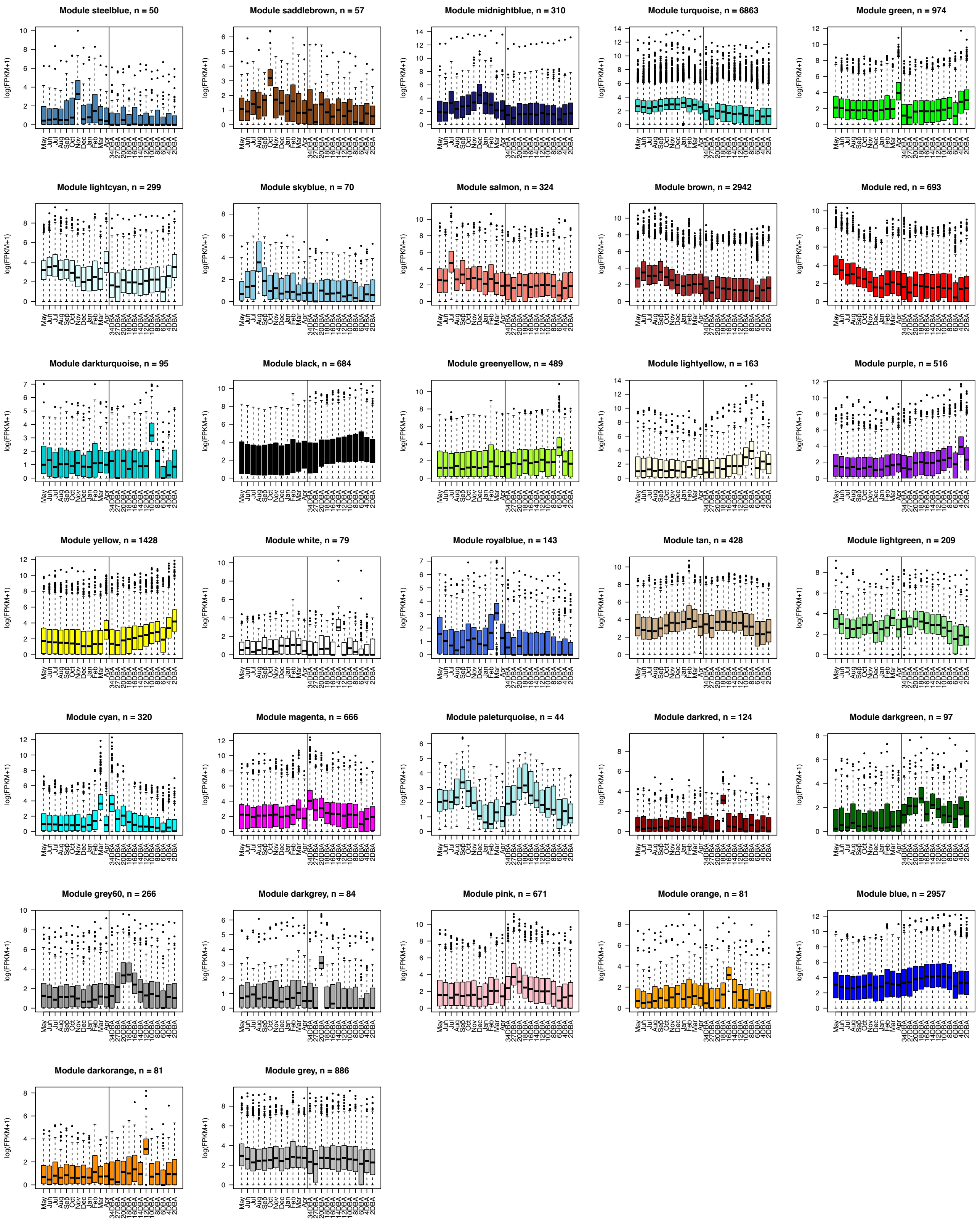

Supplementary Figure S8. Expression patterns of gene modules obtained from a weighted correlation network analysis

May to Apr are the months and 34DBA to 2DBA are days before anthesis when the bud samples were collected. Numbers of genes in each module are shown at the tops of boxplots.

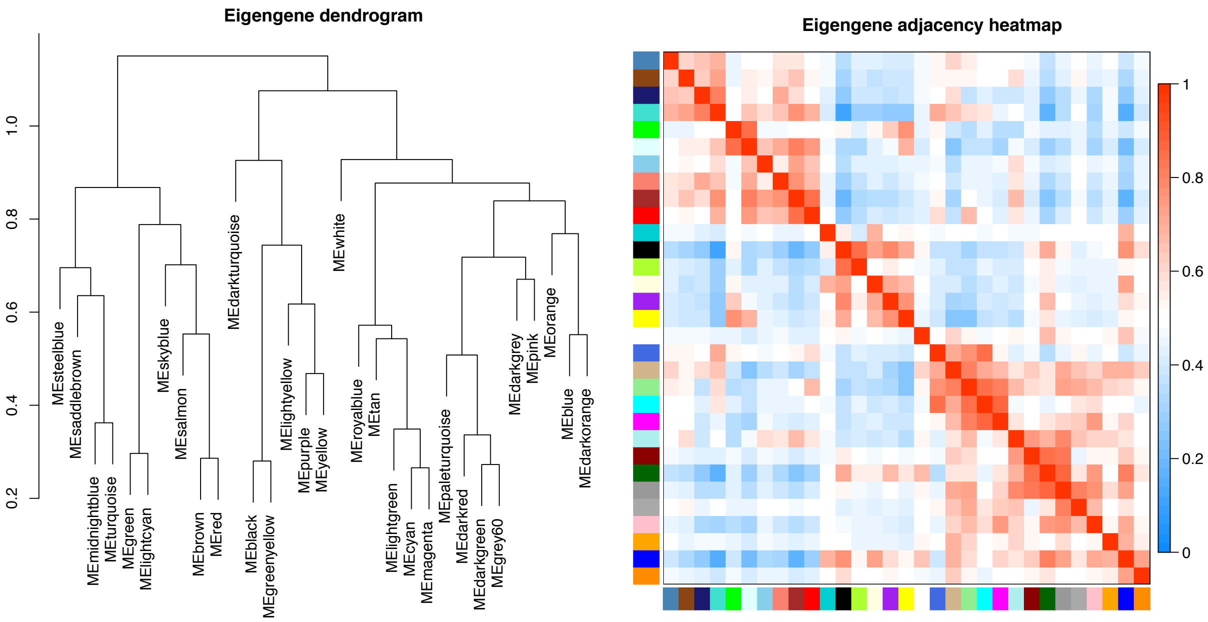

Supplementary Figure S9. Dendrogram and heatmap of gene modules represented by eigengenes obtained from a weighted correlation network analysis

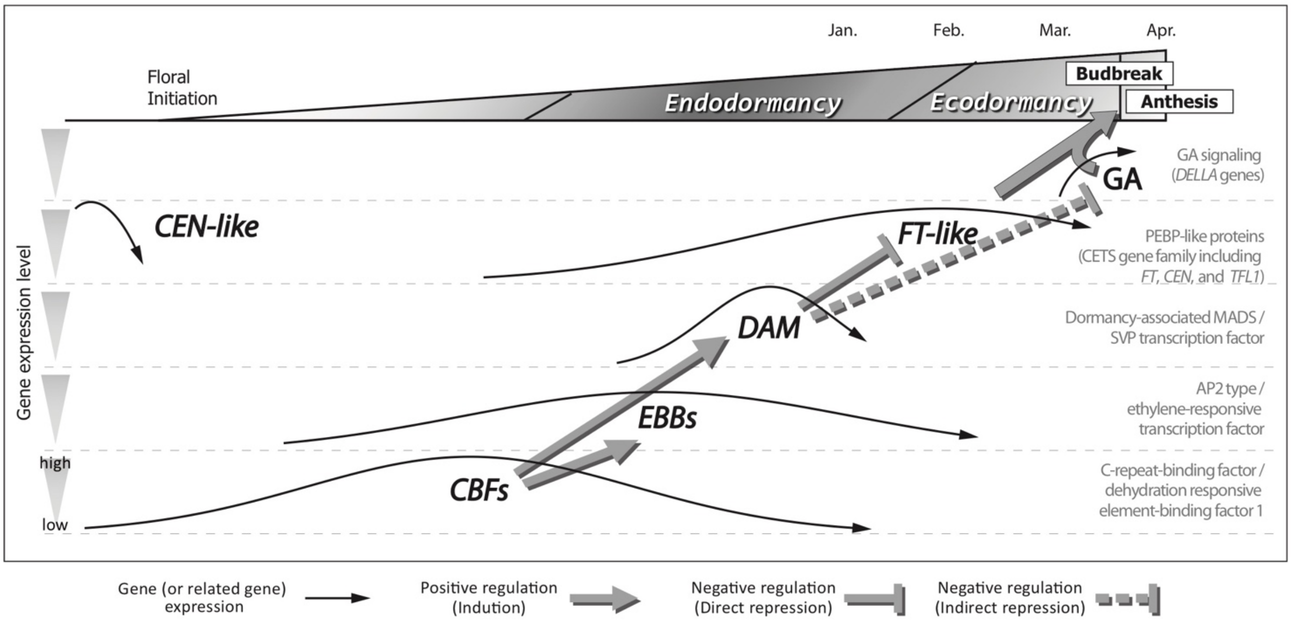

Supplementary Figure S10. A putative regulation model for dormancy release and flowering with expression patterns of related genes in ‘Somei-Yoshino’ buds

The supposed regulation mechanism for dormancy and flowering is based on recent studies and reviews in woody plants [10,73,74]. The gene expression patterns represented as black arrows are based on Figure 3.
